# Supplementary figures and images for: Overcoming Hypoxia-Induced Chemoresistance in Cancer Using a Novel Glycoconjugate of Methotrexate
Source: Pharmaceuticals (Basel). 2020 Dec 24;14(1):13. doi: 10.3390/ph14010013 (PMC7830245; doi:10.3390/ph14010013)

**a**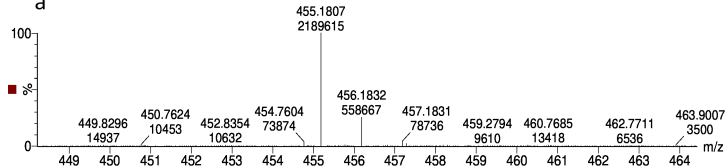**b**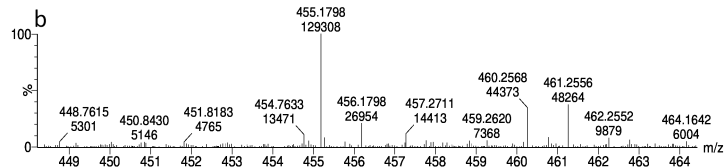

Supplement: Supplementary file 1 [file pharmaceuticals-14-00013-s001.pdf]
